# *ECRG4 *is a candidate tumor suppressor gene frequently hypermethylated in colorectal carcinoma and glioma

**DOI:** 10.1186/1471-2407-9-447

**Published:** 2009-12-17

**Authors:** Silke Götze, Valeska Feldhaus, Thilo Traska, Marietta Wolter, Guido Reifenberger, Andrea Tannapfel, Cornelius Kuhnen, Dirk Martin, Oliver Müller, Sonja Sievers

**Affiliations:** 1 Knappschaftskrankenhaus Bochum-Langendreer, Bochum, Germany; 2 Institut für Neuropathologie, Heinrich-Heine-Universität, Düsseldorf, Germany; 3 Institut für Pathologie, Universitätsklinik Bergmannsheil, Bochum, Germany; 4 Institut für Pathologie, Clemens-Hospital Münster, Germany; 5 Evangelisches Krankenhaus Witten, Klinik für Allgemein- und Viszeralchirurgie, Witten, Germany; 6 Fachhochschule Kaiserslautern, Zweibrücken, Germany; 7 Max-Planck-Institut für molekulare Physiologie, Dortmund, Germany

## Abstract

**Background:**

Cancer cells display widespread changes in DNA methylation that may lead to genetic instability by global hypomethylation and aberrant silencing of tumor suppressor genes by focal hypermethylation. In turn, altered DNA methylation patterns have been used to identify putative tumor suppressor genes.

**Methods:**

In a methylation screening approach, we identified *ECRG4 *as a differentially methylated gene. We analyzed different cancer cells for *ECRG4 *promoter methylation by COBRA and bisulfite sequencing. Gene expression analysis was carried out by semi-quantitative RT-PCR. The *ECRG4 *coding region was cloned and transfected into colorectal carcinoma cells. Cell growth was assessed by MTT and BrdU assays. ECRG4 localization was analyzed by fluorescence microscopy and Western blotting after transfection of an *ECRG4-eGFP *fusion gene.

**Results:**

We found a high frequency of *ECRG4 *promoter methylation in various cancer cell lines. Remarkably, aberrant methylation of *ECRG4 *was also found in primary human tumor tissues, including samples from colorectal carcinoma and from malignant gliomas. *ECRG4 *hypermethylation associated strongly with transcriptional silencing and its expression could be re-activated *in vitro *by demethylating treatment with 5-aza-2'-deoxycytidine. Overexpression of *ECRG4 *in colorectal carcinoma cells led to a significant decrease in cell growth. In transfected cells, ECRG4 protein was detectable within the Golgi secretion machinery as well as in the culture medium.

**Conclusions:**

*ECRG4 *is silenced via promoter hypermethylation in different types of human cancer cells. Its gene product may act as inhibitor of cell proliferation in colorectal carcinoma cells and may play a role as extracellular signaling molecule.

## Background

Genetic and epigenetic events work in concert to transform a normal cell into a malignant cancer cell. Epigenetic alterations in cancer include changes in chromatin structure and in methylation of cytosine residues in the DNA [1]. In comparison with normal cells, the cancer cell genome is hypomethylated which leads to genomic instability through the activation of mobile genetic elements [2]. Concurrently, CpG-rich regions located in many gene promoters may become hypermethylated. The hypermethylation of these 5'-CpG islands may cause transcriptional silencing of genes, including tumor suppressor genes [3]. Indeed, promoter hypermethylation often acts as second hit in the inactivation of tumor suppressor genes, e.g. when the first allele is deleted [4].

The causes of aberrant DNA methylation in cancer are not fully understood. There are hints that overexpression of DNA methyltransferases (DNMTs) may be involved, such as aberrant activity of DNMT3b promoted *de novo *methylation [5]. Moreover, higher levels of *DNMT1 *mRNA have been detected in colorectal and stomach cancers as compared to the corresponding non-neoplastic mucosa [6]. Recently, a model has been proposed to explain the phenomenon why some genes become methylated during carcinogenesis while others do not. A targeting mechanism may predispose genes that are repressed by polycomb proteins in normal cells to aberrant DNA methylation in cancer [7,8].

In many tumors, DNA methylation changes tend to accumulate with tumor progression [9]. The profiling of diverse cancer specimens has shown that each tumor type bears a specific DNA methylation pattern. Such a pattern may be used for diagnostic or prognostic purposes [10]. Moreover, aberrant DNA methylation patterns in cancer have been used for the discovery of candidate tumor suppressor genes; e.g. the *HIC-1 *(*hypermethylated in cancer*) gene was identified on chromosomal band 17p13.3, which had been described to be aberrantly methylated [11]. Subsequently, the role of *HIC-1 *in carcinogenesis as a transcriptional repressor has been elucidated [12]. A variety of methods for the genome-wide screening of methylation differences between tumor and normal cells have been established [13] and many aberrantly methylated genes have been identified in different tumor types [14]. As there are additional mechanisms of epigenetic gene regulation, DNA methylation is sometimes not tightly associated with transcriptional silencing [15,16]. Some genes even become repressed upon demethylation [17]. Thus, after identification of a gene differentially methylated in cancer versus normal tissue, its functional role in carcinogenesis remains to be proven by additional molecular and functional analyses.

In the course of a DNA methylation screening in sarcoma cells using a methylated CpG island amplification coupled with representational difference analysis (MCA-RDA) approach, we identified the *ECRG4 *gene at 2q12.2 as a gene showing aberrant promoter methylation in tumor but not in normal cells. Previous studies reported on promoter hypermethylation and reduced expression of *ECRG4 *in advanced esophageal and prostate carcinomas [18,19] and on a tumor suppressor function of ECRG4 in eosophageal cancer cell lines [20]. Here, we report on a further molecular and functional characterization of *ECRG4 *as a potential tumor suppressor gene in different types of cancer.

## Methods

### Tumor tissue samples

Thirty-one tissue samples of colorectal carcinoma and neighboring normal muscosa were analyzed. All patients and control donors provided their informed oral and written consent. Tumors were diagnosed and surgically removed in the Knappschaftskrankenhaus Bochum-Langendreer, the Universitätsklinik Bergmannsheil Bochum or the Katholisches Krankenhaus Dortmund-West between 1998 and 2003. The surgically removed tumors were classified according to the TNM/UICC system [21].

Seventy-one human glioma tissue samples, including 16 diffuse astrocytomas WHO grade II (A), 15 anaplastic astrocytomas WHO grade III (AA), 10 secondary and 30 primary glioblastomas WHO grade IV (sGBM and pGBM), were collected at the Department of Neuropathology, Heinrich-Heine-University, Düsseldorf. All samples were analyzed in an anonymized manner as approved by the local institutional review boards. Histological classification was performed according to the WHO classification of tumors of the nervous system [22]. As non-neoplastic reference tissue, we used commercially available adult human brain DNA (BD Biosciences, St. Jose, USA) as well as DNA extracted from a cerebral tissue sample of one adult patient who was operated on for a non-neoplastic lesion.

### Methylated CpG island amplification coupled with representational difference analysis (MCA-RDA)

MCA-RDA was carried out according to Toyota et al. [14]. Pooled tumor DNA from three male patients with G3 myxoid-round cell liposarcoma (round cell component > 60%) was used as tester DNA. Pooled genomic DNA from fat tissue of three male patients with non-neoplastic disease was used as driver DNA. Briefly, 5 μg of driver and tester DNA were sequentially digested with SmaI and XmaI. Following adapter ligation, both samples were PCR amplified using FastStart Taq DNA Polymerase (Roche, Mannheim, Germany). Adapter sequences were then removed from the driver and tester amplicon. New adapter sequences were ligated only on the tester amplicon. After two rounds of competitive hybridization and selective amplification, the MCA products were cloned into the pDrive vector (Qiagen, Hilden, Germany). Next, 193 clones were amplified by PCR and analyzed by dot-blot hybridization using an Alu probe. Non-Alu hybridizing inserts were sequenced and mapped onto the human genome using BLAT and BLAST [23,24]. Amongst others, a fragment of the *ECRG4 *gene, corresponding to a part of intron 1 was cloned (Chr. 2:106682377-106682546; UCSC genome browser, February 2009 built). The genomic region of the *ECRG4 *gene including 1000 bp upstream of the transcription start site was analyzed for its CpG content using Methprimer [25] and a 413 bp CpG island was identified.

### Cell culture

Eleven tumor cell lines from various tissues (Tab. 1) were obtained from American Type Culture Collection (ATCC, Manassas, USA) and cultured according to standard protocols. To reverse methylation of the *ECRG4 *promoter, cell lines were treated with 0 to 10 μM 5-aza-2'-deoxycytidine (AZA) for three to four days. In addition, trichostatin A (TSA) treatment was carried out alone or in combination with AZA treatment at a concentration of 1 μM for 24 h. AZA and TSA were purchased from Sigma (Munich, Germany).

**Table 1:** Cancer cell lines analyzed for *ECRG4 *hypermethylation and expression

cell line	tumor type
A549	lung carcinoma [36]

HCT116	colorectal carcinoma [37]

HeLa	cervical adenocarcinoma

HepG2	hepatocellular carcinoma [38]

HT-29	colorectal adenocarcinoma [39]

HT1080	fibrosarcoma [40]

MCF7	breast adenocarcinoma [41]

SW480	colorectal adenocarcinoma [42]

U87-MG	glioblastoma [43]

U373-MG	glioblastoma [43]

T98G	glioblastoma [44]

### DNA isolation and sodium bisulfite modification

DNA from fresh frozen colorectal tumor tissue samples and from cell lines was isolated with the QIAamp DNA Mini Kit (Qiagen). DNA from glioma tissue samples was extracted by ultracentrifugation over caesium chloride as reported elsewhere [26]. Methylated control DNA was prepared by treatment of human lymphocyte DNA with *M. Sss*I methylase (New England Biolabs, Ipswich, USA). Bisulfite modification of DNA was carried out with the EZ DNA Methylation-Gold Kit (Zymo Research, Orange, USA) according to the manufacturer's instructions.

### Methylation analysis of the ECRG4 promoter region

**Figure 1:**
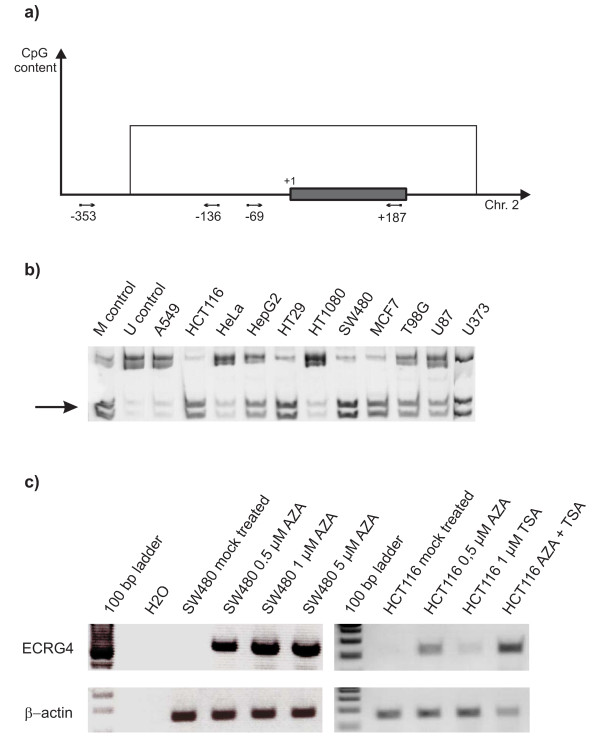
***ECRG4 *promoter methylation and expression in different cancer cell lines**. a) Schematic representation of the 5'-CpG island located in the *ECRG4 *promoter region and covering exon 1 as well as parts of intron 1. Primer binding sites are indicated by arrows and labeled with numbers. The transcription start site is marked with +1; the first exon is represented by the grey box. b) COBRA analysis of *ECRG4 *promoter methylation in various cancer cell lines. The arrow indicates the fragments corresponding to the methylated sequence. c) Re-expression of *ECRG4 *in SW480 and HCT116 cells following treatment with AZA at different concentrations, either alone or in combination with TSA as indicated in the figure.

The *ECRG4 *promoter region contains a 5'-CpG island covering the promoter region, exon 1 and parts of intron 1 (Fig. 1a). Methylation of an upstream (-353/-136 relative to transcription start site) and a downstream (-69/+187 relative to transcription start site) fragment from this CpG island was determined by COBRA (combined bisulfite and restriction analysis). Unless otherwise stated, all PCR reactions were performed using 1.5 mM MgCl_2_, 0.2 mM of each dNTP, 0.3 μM of each primer and 0.5 units *HotStarTaq *DNA polymerase (Qiagen). Following an initial denaturation step for 15 min at 95°C, cycling conditions were 40 cycles for 30 s at 95°C, 30 s at the specified annealing temperature (T_a_) and 30 s at 72°C; followed by a final 10 min elongation step at 72°C. The upstream promoter region was amplified from sodium bisulfite treated DNA using primers -353: 5'-AGTGGGGGAGTTAAGGAGATATTTT-3' and -136: 5'-CTAAACTCCAAAACCAAAAATACTTAA-3' at a T_a _of 63°C. The resulting 217 bp product was digested into a 56 bp and a 161 bp product by incubation with *BstU*I. The downstream promoter region was amplified using primers -69: 5'-GTAGT(C/T)GTTTGGTTTTTAGTTTTT-3' and +187: 5'-CTAAACCCCAACACAAAAACAAA-3' yielding a 256 bp product which was digested with *Taq*I resulting in a 118 bp and a 138 bp product. Digestion products were separated by polyacrylamide gel electrophoresis (8%) and visualized by ethidium bromide staining. COBRA analysis was carried out at least in duplicate.

PCR products (upstream fragment) obtained from sodium bisulfite-modified DNA of selected cell lines were cloned into the *pDrive *vector (Qiagen) and ten individual clones were sequenced. The sequences were analyzed using the BiQ Analyzer program [27]; conversion rates were found to be between 96% and 100%.

PCR products (downstream fragment) amplified from sodium bisulfite-modified of selected tumor samples were purified and methylation percentages were determined by Epitect sequencing (Qiagen). The Epitect sequencing service includes sequencing of the PCR products using cycle sequencing and modified analysis of raw data to obtain a percentage of methylation for each CpG dinucleotide. Nineteen CpG sites in the promoter fragment were successfully analyzed; the methylation percentages of these sites were averaged to obtain a single methylation score for each sample. Samples with a methylation score three times higher than normal colon tissue were considered as being methylated. As the four analyzed normal colon samples showed an averaged methylation score of 3.7% a methylation score higher than 11.1% was considered as being methylated.

### Analysis of ECRG4 expression by reverse transcription PCR

RNA from fresh frozen colorectal tumor tissues and cell lines was isolated using the NucleoSpin RNA II Kit (Macherey-Nagel, Düren, Germany). One microgram of RNA was reverse transcribed using the Revert Aid H-Minus M-Mulv RT Kit (Fermentas, Vilnius, Lithuania). In the cell lines, a fragment of the *ECRG4 *cDNA spanning all exons was amplified using primers ECRG4_RT_ for: 5'-GGTTCTCCCTCGCAGCACCT-3' and ECRG4_RT_rev: 5'-CAGCGTGTGGCAAGTCATGGTTAGT-3' at a T_a _of 61°C (527 bp). As this long fragment was difficult to obtain in tumor samples, a shorter fragment of *ECRG4 *was amplified using forward primer ECRG4_RT_for2: 5'-GGTACCAGCAGTTTCTCTACATG-3' and the reverse primer stated above at a T_a _of 62°C (220 bp). As internal control, a 156 bp fragment of the *β-actin *gene was amplified using primers ACTIN_RT_for: 5'-TCATGAAGTGTGACGTGGACATC-3' and ACTIN_RT_rev: 5'-CAGGAGGAGCAATGATCTTGATCT-3' at a T_a _of 60°C. For semiquantitative expression analysis, the *ECRG4 *RT-PCR was carried out for 33 cycles and the *β-actin *RT-PCR for 26 cycles. PCR products were separated on a 2% agarose gel and visualized by ethidium bromide staining. Band intensities (BI) were quantified using the AIDA image analyzer program (Raytest, Straubenhardt, Germany) and relative expression levels were calculated as BI(*ECRG4*)/BI(*β-actin*).

### Cloning of ECRG4 constructs

The complete *ECRG4 *coding region was cloned into the pCI Neo vector (Promega, Madison, USA) for transient transfection of cancer cell lines. The coding region was amplified from cDNA of AZA-treated HCT116 cells using primers 5'-gctagCATGGCTGCCTCCCCCGC-3' and 5'-tctagaTTAGTAGTCATCGTAGTTGACGC-3' which contain restriction site tags for *Nhe*I and *Xba*I, respectively (typed in lower letters). The *ECRG4 *coding region was also cloned into the peGFP-N1 vector (Clontech, Mountain View, USA) with primers 5'-gctagCATGGCTGCCTCCCCCGC-3' and 5'-gctagcATGCTTCAAAAACGAGAAGCACC-3' which contain restriction site tags for *Nhe*I and *Hind*III, respectively. As controls, cells were transfected with the empty vectors.

### Production of ECRG4 containing medium

For the production of ECRG4 or ECRG4-eGFP containing medium, 2 × 10^6 ^HCT116 cells were plated in 10 cm dishes. The next day, cells were transiently transfected with the ECRG4-pCI Neo or ECRG4-eGFP and control vectors, respectively, using 15 μl Attractene (Qiagen) and 4 μg of plasmid DNA. After twenty-four hours, the cell culture medium was replaced with fresh medium without additives. Again twenty-four hours later, the cell culture medium was collected and "Complete" protease inhibitor cocktail (Roche) was added. Cellular debris was removed by centrifugation at 700 g and the medium was concentrated using Amicon-15 centrifugal filter units (Millipore, Billerica, USA). Concentrated medium was used directly or was stored at 4°C.

### Cell proliferation assays

Cell growth and viability were evaluated by using BrdU and MTT assays, respectively. For the BrdU assay, 6 × 10^4 ^HCT116 or SW480 cells were plated in quadruplicate in 96-well plates. The next day, ECRG4-pCI Neo and control plasmids were transfected using 0.75 μl Attractene (Qiagen) and 0.2 μg of plasmid DNA. Forty-eight hours post transfection, the BrdU assay (BrdU cell proliferation ELISA; Roche) was carried out according to the manufacturer's instructions. For the MTT assay, cells were plated and transfected as described above. Forty-eight hours post transfection, MTT solution (5 mg/ml 3-(4,5-Dimethylthiazol-2-yl)-2,5-diphenyltetrazolium bromide in PBS) was added and cells were incubated for 2 h at 37°C. Then 0.04 N HCl in 2-propanol was added and, after 45 min, the absorption was measured photometrically at 550 nm with a background subtraction at 690 nm. For both assays, the mean absorbance values of at least three independent experiments were used for statistical analysis.

The MTT assay was also carried out after treatment of cells with ECRG4 containing medium and medium from cells transfected with pCI Neo. Therefore, the cells were plated as described. The next day, the cell culture medium was removed and 50 μl of ECRG4 containing medium was added. After 48 hours, the MTT assay was carried out as described.

### Western blot analysis

For the detection of the ECRG4-eGFP fusion protein, cellular extracts or medium proteins were separated on a 20% SDS-polyacrylamide gel and blotted. Immunodetection was carried out using an eGFP antibody (Abcam, Cambridge, UK) after over night incubation at a dilution of 1:2000 in TBS with 0.1% Tween 20 and 5% skimmed milk powder.

### Fluorescence microscopy

For cellular localization of *ECRG4*-eGFP fusion proteins, cells were grown on cover slips, transiently transfected with the ECRG4-peGFP-N1 or control vectors using 1.5 μl Attractene (Qiagen) and 0.4 μg of plasmid DNA. Beta-1,4-galactosyltransferase (GalT), a Golgi resident protein, was used as Golgi marker. Therefore, a GalT-GFP construct was transfected as described. Forty-eight hours after transfection, cells were counterstained with DAPI and analyzed by fluorescence microscopy at 400-fold magnification.

### Statistical analysis

Statistical calculations were carried out with the GraphPad QuickCalcs online calculator http://www.graphpad.com/quickcalcs and STATISTICA 8 software (StatSoft, Hamburg, Germany). A p-value of less than 0.05 was considered as indicating statistical significance.

## Results

To identify newly hypermethylated genes in cancer, we performed a methylated CpG island amplification coupled with representational difference analysis (MCA-RDA) approach, slightly modified to Toyota et al. [14], using soft tissue sarcoma and control tissues (data not shown). Among several candidate sequences, a fragment of the *ECRG4 *promoter was cloned.

### ECRG4 promoter methylation and expression in tumor cell lines

To elucidate the role of *ECRG4 *hypermethylation in human tumor cells, we analyzed the methylation of its promoter in eleven cancer cell lines from colorectal, lung, cervix, hepatocellular and breast carcinoma, fibrosarcoma and glioblastoma (Table 1). COBRA analysis of the upstream promoter fragment showed either full or partial methylation in all cell lines. To confirm these results, sodium bisulfite sequencing of PCR products from HCT116 and SW480 cells was carried out and also showed dense promoter methylation in both colorectal carcinoma lines (data not shown). However, we wondered if the methylation of the *ECRG4 *promoter extends further downstream towards the transcription start site. We therefore analyzed a second promoter fragment by COBRA and found that all three colorectal carcinoma and all three glioblastoma cell lines, as well as HepG2 and MCF7 were methylated whereas A549, HeLa and HT1080 were not (Fig. 1b).

We analyzed the panel of tumor cell lines for expression of *ECRG4 *using RT-PCR. In these analyses, the *ECRG4 *transcript was not detected even after 40 cycles of amplification. *ECRG4 *was re-expressed in the colon cancer cell lines SW480, HCT116, HT-29 and the fibrosarcoma cell line HT1080 after treatment with the demethylating agent AZA. Treatment with TSA alone had no effect. Combined treatment with AZA and TSA induced strong expression (Fig. 1c and data not shown).

### ECRG4 promoter methylation and expression in colon carcinoma

**Figure 2:**
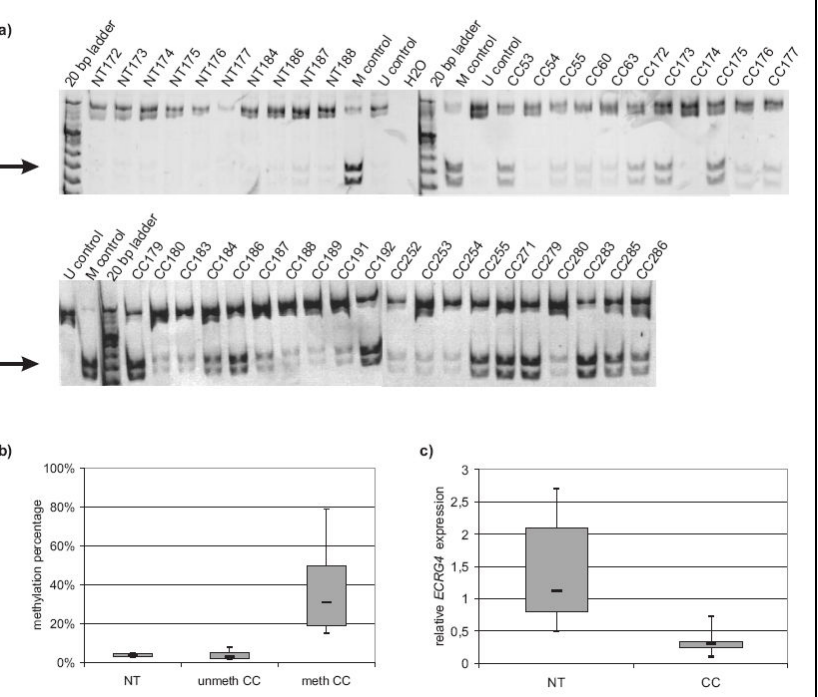
***ECRG4 *promoter methylation and mRNA expression in primary colorectal carcinoma samples**. a) COBRA analysis of *ECRG4 *methylation in colorectal carcinoma and normal colon tissue samples. Arrows point to restriction fragments indicating a methylated sequence. b) Box plots of methylation percentages of selected colorectal carcinomas and normal colon tissue as determined by Epitect bisulfite sequencing analysis. unmeth = unmethylated; meth = methylated c) Box plots of relative *ECRG4 *mRNA expression as determined by semiquantitative RT-PCR in colorectal carcinomas relative to normal colon tissue. Note a significantly lower expression in tumor tissue (p = 0.0018). NT = normal colon tissue; CC = colorectal carcinoma.

As the three analyzed colon cancer cell lines showed *ECRG4 *hypermethylation, we asked whether *ECRG4 *methylation also can be found in primary colorectal tumors. We therefore analyzed 31 samples from colon carcinoma and 10 corresponding normal tissues by COBRA (downstream fragment). While tumor samples showed a high methylation frequency (19 of 31; 61%), control tissues lacked detectable methylation (0/10; 0%) (Fisher's exact test; p = 0.023) (Fig. 2a). Results of selected samples were validated using the Epitect Sequencing Service (Qiagen). Eight methylated samples displayed methylation levels ranging from 15% to 79%, seven unmethylated samples showed methylation levels ranging from 2% to 8% and four normal colon tissues showed methylation levels of 3% to 5% (Fig. 2b). Thus, qualitative and semiquantitative methylation analysis revealed a significantly higher methylation frequency in colon carcinoma as compared to neighboring colorectal mucosa (unpaired t-test; p = 0.0166). To determine if *ECRG4 *promoter methylation is correlated to gene expression, we analyzed relative expression levels of *ECRG4 *by semiquantitative RT-PCR in eight tumor and nine normal tissues. Of the eight tumor tissues, six were hypermethylated and two were of unknown methylation status. We found significantly lower expression of *ECRG4 *in tumor tissues as compared to control tissue (BI = 0.33 vs. BI = 1.44; unpaired t-test; p = 0.0018) (Fig. 2c).

### ECRG4 promoter methylation in glioma

**Figure 3:**
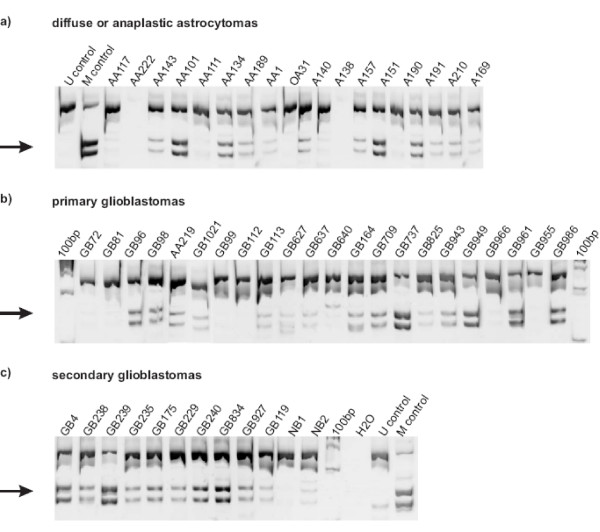
***ECRG4 *promoter methylation in primary glioma tissue samples**. COBRA analysis of *ECRG4 *methylation in astrocytic gliomas of different WHO grades and two non-neoplastic brain samples (NB1, NB2). Representative data from 49 samples are shown. Arrows point to restriction fragments indicating a methylated sequence. The diagnosis is given in the subheadings. The tumor numbers are provided on top of each lane.

Next, we analyzed 71 glioma samples for methylation of the *ECRG4 *promoter by COBRA. Seven out of 31 diffuse or anaplastic astrocytomas (23%), 12 out of 30 primary (40%) and 9 out of 10 secondary (90%) glioblastomas carried a methylated *ECRG4 *promoter (Fig. 3). In contrast, the two investigated normal brain samples lacked *ECRG4 *promoter methylation. Statistical analysis revealed that *ECRG4 *methylation was significantly more frequent in secondary glioblastomas as compared to diffuse and anaplastic astrocytomas or primary glioblastomas (Fisher's exact test; p = 0.0003 and p = 0.0094, respectively).

### Effect of ECRG4 protein on colorectal carcinoma cell proliferation

**Figure 4:**
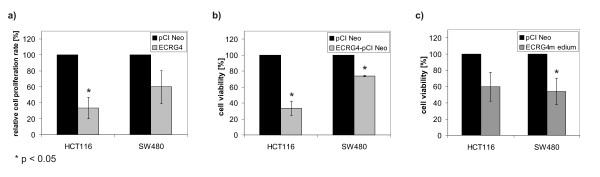
**Effects of *ECRG4 *overexpression or ECRG4 treatment in colorectal carcinoma cells on proliferation and viability**. a) Reduced proliferation rate of *ECRG4 *transfected HCT116 and SW480 cells relative to control cells as determined by the BrdU assay (n = 3; p = 0.0133) (n = 3; p = 0.0775). b) Reduced cell viability of *ECRG4 *transfected HCT116 and SW480 cells relative to control cells as determined by the MTT assay (n = 4; p = 0.0005) (n = 3; p = 0.0003). c) Reduced cell viability of HCT116 and SW480 cells treated with ECRG4-containing medium relative to cells treated with medium from pCI Neo transfection as determined by the MTT assay (n = 3; p = 0.0603) (n = 3; p = 0.0397).

To evaluate the cellular effects of re-expression of *ECRG4*, we cloned the *ECRG4 *coding region and transiently transfected the gene into the colorectal carcinoma cell lines HCT116 and SW480. The proliferation rate of *ECRG4 *transfected HCT116 cells, as assessed by the BrdU assay, was significantly reduced to 33% (± 13%; one sample t-test; p = 0.0133) relative to the mock-transfected control cells (Fig. 4a). In the MTT assay, relative cell viability of *ECRG4 *transfected HCT116 and SW480 cells was significantly reduced to 33% (± 9%; one sample t-test; p = 0.0005) and 74% (± 1%; one sample t-test; p = 0.0003) (Fig. 4b), respectively. To eliminate the influence of the transfection on tumor cell growth, we measured cell viability after treatment of cells with ECRG4 containing medium and medium from transfection with pCI Neo. Again, ECRG4 treated HCT116 and SW480 cells showed a reduced cell viability relative to control treated cells (60% ± 18%; one sample t-test; p = 0.0603) (54% ± 16%; one sample t-test; p = 0.0397) (Fig. 4c). Thus, re-expression of the silenced *ECRG4 *gene as well as treatment of cells with ECRG4 protein significantly reduced colon cancer cell growth.

### Localization of ECRG4 in cultured colorectal carcinoma cells

**Figure 5:**
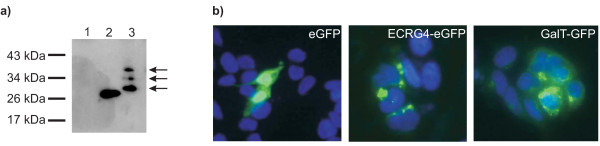
**Extracellular secretion and subcellular localization of the ECRG4 protein**. a) Western Blot analysis with an anti-GFP antibody of concentrated cell culture medium of ECRG4-eGFP transfected HCT116 cells shows that ECRG4 is secreted. 1 = concentrated medium of non-transfected cells; 2 = total lysate of eGFP transfected cells; 3 = concentrated medium of ECRG4-eGFP transfected cells. Arrows point to three different processed forms of the ECRG4-eGFP fusion protein. b) Fluorescence microscopy analysis of HCT116 cells transfected with eGFP, ECRG4-eGFP and GalT-GFP vectors. Note that transfection with the eGFP control vector leads to homogenous staining, whereas cells transfected with the ECRG4 fusion gene show staining of discrete structures. These structures are also stained after transfection of the GFP tagged Golgi marker galactosyltransferase (GalT-GFP).

Finally, we analyzed the subcellular localization of the ECRG4 protein and its possible secretion into the culture medium. Therefore, we transfected an *ECRG4-eGFP *fusion gene into HCT116 cells. The cell culture medium was collected 48 h post-transfection and concentrated. Western Blotting with an eGFP antibody detected the ECRG4-eGFP fusion protein as three distinct bands of ~40 kDa, ~35 kDa and ~28 kDa (Fig. 5a). The highest molecular weight (MW) band would fit to the MW of the ECRG4-eGFP fusion protein (eGFP: 26 kDa + ECRG4 without signal peptide: 14 kDa). The lower MW bands presumably represented ECRG4-eGFP proteins with N-terminally processed ECRG4.

We also analyzed the intracellular localization of ECRG4 by fluorescence microscopy after transfection of the fusion gene. Whereas cells transfected with the eGFP vector control showed homogenous staining all over the cell, the ECRG4-eGFP transfected cells displayed discrete staining localized in close proximity to the nucleus (Fig. 5b). This staining pattern was also obtained after transfection of cells with a GFP tagged construct of the golgi resident protein β-1,4-galactosyltransferase. Thus, it could be concluded that ECRG4 was localized to the Golgi apparatus which would be in accordance with the secretory pathway of proteins.

## Discussion and Conclusions

The data reported in this study implicate *ECRG4 *as a candidate tumor suppressor gene at 2q12.2 that is frequently hypermethylated and transcriptionally down-regulated in colon carcinomas and other types of human cancers. Cancer cells are characterized by extensive epigenetic DNA alterations, including global hypomethylation as well as focal hypermethylation at CpG islands in gene promoters. DNA hypermethylation constitutes a major cause of aberrant gene silencing in cancer [28]. However, not all aberrantly silenced genes also play functional roles in tumor development. In analogy to somatic mutations in tumor cells, which in part are neutral passenger mutations, some methylation events might be epigenetic passengers [29]. Therefore, each candidate tumor suppressor gene identified by methylation screening methods has to be carefully evaluated for its role in tumor development and progression.

In the course of a methylation screening approach, we identified *ECRG4 *as a gene differentially methylated in sarcoma versus normal cells. Previously, *ECRG4 *had been found to be hypermethylated in esophageal cancer [30]. To investigate its possible role in human cancer, we (1) analyzed different tumor entities for *ECRG4 *promoter methylation, (2) revealed a correlation between the methylation and gene silencing, (3) measured the effect of *ECRG4 *re-expression on the proliferation of colon cancer cells, and (4) determined the cellular localization of the ECRG4 protein.

Initially, we found the *ECRG4 *promoter to be frequently hypermethylated in a broad spectrum of human tumor cell lines. To exclude that hypermethylation is limited to tumor cells in culture we carried out qualitative and semiquantitative methylation analysis of primary cancer samples. In colorectal carcinomas, we found a high frequency of methylation, but, as the analyzed samples were all from advanced staged tumors, correlations to clinical features were not possible. We also found frequent methylation in astrocytic gliomas, in which the methylation frequency increased with advancing tumor grade, being particularly common in glioblastomas. This result is consistent with recent reports that *ECRG4 *methylation is associated with advanced disease and poor prognosis in esophageal and prostate cancer patients [18,31]. Our findings of frequent *ECRG4 *methylation in various cancer cell lines may suggest that *ECRG4 *might be methylated in even other tumor types. Hence, *ECRG4 *might belong to the set of genes that are frequently methylated in multiple types of cancer. Another example is the pro-apoptotic gene *RASSF1A*, which is rarely mutated but often hypermethylated in various kinds of epithelial, mesenchymal and neuroectodermal tumors [32]. Such genes often regulate processes that are crucial for cancer development, such as apoptosis or cell cycle progression.

Our data also indicate that *ECRG4 *promoter methylation strongly correlates with transcriptional silencing. None of the methylated cancer cell lines showed expression of *ECRG4 *transcripts. Furthermore, the expression level of *ECRG4 *was significantly lower in colon cancer samples than in normal mucosa, which agrees with the recent result that *ECRG4 *expression is lower in esophageal cancer than in surrounding normal epithelium [19]. It should be noted that two normal colon tissues also showed weak *ECRG4 *expression. This could be due to a methylation field effect in normal tissue surrounding tumor tissue. Alternatively, it cannot be ruled out that *ECRG4 *silencing might be regulated by other mechanisms than DNA methylation. Then again, *ECRG4 *expression could be reactivated by demethylating treatment, which is a known feature of epigenetically silenced tumor suppressor genes [33,34].

By *ECRG4 *overexpression or ECRG4 treatment, we demonstrate an inhibitory effect of this protein on colorectal carcinoma cell growth. Using two independent assays of cell viability and cell proliferation, we found reduced growth rates after *ECRG4 *transfection or culturing of cells in ECRG4 containing medium. An additional hint supporting a growth suppressive effect of *ECRG4 *in tumor cells comes from stabile transfection experiments, in which we observed that the expression of the transgene rapidly declined, presumably by *de novo *methylation (data not shown). Recent findings show that ECRG4 acts as a tumor suppressor in esophageal cancer cells in vitro and in vivo [20]. Our data provide first hints that ECRG4 may serve a tumor suppressor function also in colorectal carcinoma cells.

Finally, we demonstrate that ECRG4 is a secreted protein. The *ECRG4 *gene codes for a small protein of 148 aa (17 kDa) of yet unknown function and without significant homology to other known proteins, except for a 31% homology to the mouse IgG V region [19]. A putative N-terminal signal peptide suggests that ECRG4 might be a secreted protein. We found that an ECRG4-eGFP fusion protein localized to the Golgi apparatus within the cell, which would be consistent with the secretory pathway of proteins. In addition, we detected the ECRG4-eGFP fusion protein in the cell culture medium of transfected cells. Recently, the ECRG4 protein was renamed in the Uniprot database http://www.uniprot.org as "augurin", a protein which had been identified in a bioinformatics approach as a putative new peptide hormone [35]. Mirabeau et al. showed that augurin is a secreted protein which is processed from its prepro-form. However, only limited augurin expression was found in murine endocrine tissues. In addition to its potential expression in endocrine glands, *ECRG4 *is expressed in normal epithelium of the colon and esophagus and in fat tissue as shown here and in other studies [18,19]. The findings of Mirabeau et al. (2007) support the hypothesis that ECRG4 functions as an extracellular signaling molecule. Interestingly, unlike other peptide hormones, the signaling of ECRG4 is not growth promoting but rather growth limiting.

In conclusion, we show that the *ECRG4 *gene is frequently hypermethylated in a variety of human cancer cells. Hypermethylation associates strongly with transcriptional silencing in colorectal carcinomas. Furthermore, ECRG4 displays growth-inhibitory effects on colon cancer cells *in vitro *and may function as a secreted extracellular signaling molecule. Thus, restoring ECRG4 expression in the tumor, either by epigenetic therapy or application of recombinant protein, may represent a promising novel therapeutic approach in various types of cancers.

## Abbreviations

AZA: 5-aza-2'-deoxycytidine; *ECRG4*: esophageal cancer related gene 4; TSA: trichostatin A;

## Competing interests

The authors declare that they have no competing interests.

## Authors' contributions

SG carried out the DNA methylation and gene expression studies and drafted the manuscript, VF carried out the cloning and the functional characterization experiments, TT analyzed colon carcinoma samples, MW and GR analyzed and interpreted the results of the glioblastoma samples and drafted the manuscript, AT analyzed colon carcinoma samples and drafted the manuscript, CK and DM analyzed colon carcinoma samples, OM participated in the design of the study and drafted the manuscript, SS conceived of the study, participated in its design and coordination and drafted the manuscript. All authors read and approved the final manuscript.

## Pre-publication history

The pre-publication history for this paper can be accessed here:

http://www.biomedcentral.com/1471-2407/9/447/prepub
